# Supplementary material for: Neutralized Dicalcium Phosphate and Hydroxyapatite Biphasic Bioceramics Promote Bone Regeneration in Critical Peri-Implant Bone Defects
Source: Materials (Basel). 2020 Feb 11;13(4):823. doi: 10.3390/ma13040823 (PMC7079663; doi:10.3390/ma13040823)
Supplement: Supplementary file 1 [file materials-13-00823-s001.pdf]

materials

Supplementary Materials

Neutralized Dicalcium Phosphate and Hydroxyapatite Biphasic Bioceramics Promote Bone Regeneration in Critical Peri-implant Bone Defects

Hao-Hueng Chang [1,2], Chun-Liang Yeh [2], Yin-Lin Wang [1,2], Kang-Kuei Fu [1], Shang-Jye Tsai [1,3], Ju-Hsuan Yang [2] and Chun-Pin Lin [1,2,*]

[1] Graduate Institute of Clinical Dentistry, School of Dentistry, National Taiwan University, Taipei 10048, Taiwan; changhh@ntu.edu.tw (H.-H.C.); wil1019@ntu.edu.tw (Y.-L.W.); allenfu@hotmail.com (K.-K.F.); shangjye707@yahoo.com.tw (S.-J.T.)
[2] Department of Dentistry, National Taiwan University Hospital, Taipei 10048, Taiwan; staryeh0524@gmail.com (C.-L.Y.); D06422001@g.ntu.edu.tw (J.-H.Y.)
[3] Department of Dentistry, Cardinal Tien Hospital Yonghe Branch, New Taipei City 23445, Taiwan
* Correspondence: chunpinlin@gmail.com

Received: 24 December 2019; Accepted: 10 February 2020; Published: 11 February 2020

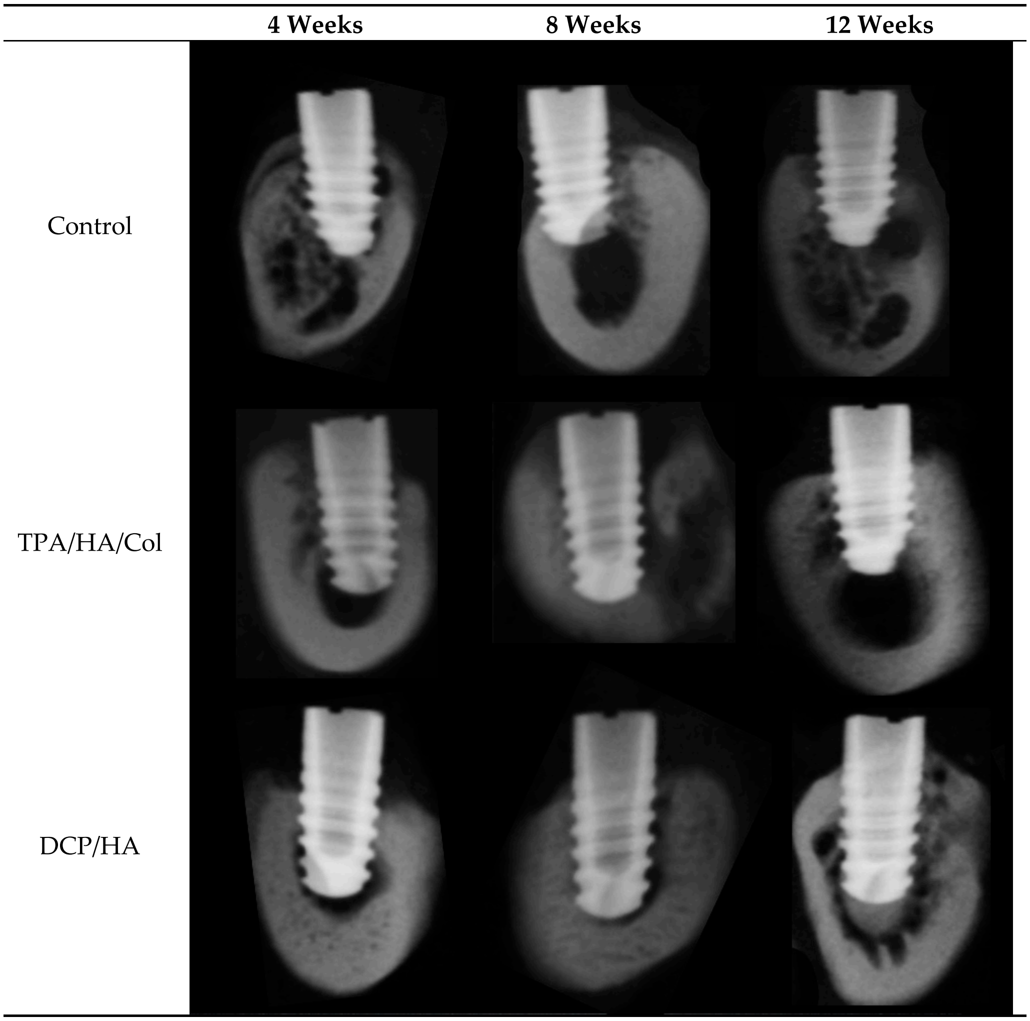

Figure S1. Radiographic images of the dental implant and surrounding bone following cross-section on the buccal–lingual plane.
